# Transcriptional regulation of respiration in yeast metabolizing differently repressive carbon substrates

**DOI:** 10.1186/1752-0509-4-12

**Published:** 2010-02-18

**Authors:** Sarah-Maria Fendt, Uwe Sauer

**Affiliations:** 1 Institute of Molecular Systems Biology, ETH Zurich, Zurich, Switzerland; 2 Life Science Zurich PhD Program on Systems Biology of Complex Diseases, Zurich, Switzerland; 3 Competence Center for Systems Physiology and Metabolic Diseases, Zurich, Switzerland

## Abstract

**Background:**

Depending on the carbon source, *Saccharomyces cerevisiae *displays various degrees of respiration. These range from complete respiration as in the case of ethanol, to almost complete fermentation, and thus very low degrees of respiration on glucose. While many key regulators are known for these extreme cases, we focus here on regulators that are relevant at intermediate levels of respiration.

**Results:**

We address this question by linking the functional degree of respiration to transcriptional regulation via enzyme abundances. Specifically, we investigated aerobic batch cultures with the differently repressive carbon sources glucose, mannose, galactose and pyruvate. Based on ^13^C flux analysis, we found that the respiratory contribution to cellular energy production was largely absent on glucose and mannose, intermediate on galactose and highest on pyruvate. *In vivo *abundances of 40 respiratory enzymes were quantified by GFP-fusions under each condition. During growth on the partly and fully respired substrates galactose and pyruvate, several TCA cycle and respiratory chain enzymes were significantly up-regulated. From these enzyme levels and the known regulatory network structure, we determined the probability for a given transcription factor to cause the coordinated expression changes. The most probable transcription factors to regulate the different degrees of respiration were Gcr1p, Cat8p, the Rtg-proteins and the Hap-complex. For the latter three ones we confirmed their importance for respiration by quantifying the degree of respiration and biomass yields in the corresponding deletion strains.

**Conclusions:**

Cat8p is required for wild-type like respiration, independent of its known activation of gluconeogenic genes. The Rtg-proteins and the Hap-complex are essential for wild-type like respiration under partially respiratory conditions. Under fully respiratory conditions, the Hap-complex, but not the Rtg-proteins are essential for respiration.

## Background

Depending on the environmental conditions, and in particular the nature of carbon substrates, *Saccharomyces cerevisiae *adjusts its energy metabolism in a process that is generally referred as carbon catabolite repression. Two extreme cases are exponential growth on glucose and on ethanol, which lead to almost exclusive fermentation with extensive secretion of ethanol and exclusive respiration to carbon dioxide, respectively.

Both, fully respiratory and fermentative energy production is mediated, to a large extent, by differentially active transcription factors. During fermentative metabolism, mainly the transcription factor complex of Tup1p, Ssn6p and Mig1p repress the expression of respiratory, gluconeogenic and alternative carbon source utilization genes [1-3]. Minimal activity of the tricarboxylic acid (TCA) cycle for biosynthetic purposes under fermentative conditions is mainly assured by the Rtg transcriptional activators [4-6]. During respiratory growth on non fermentable carbon sources, on the other hand, respiratory genes in the TCA cycle and respiratory chain are highly induced. This is triggered by the Hap transcription factors, a global activator complex of respiratory genes [1,4,6-8]. Activation of genes required for gluconeogenesis during growth on non fermentable carbon sources is achieved by the transcriptional activators Cat8p and Sip4p [3,4,9,10]. These are the key elements of the yeast transcriptional regulatory network for the extreme cases of fermentative and fully respirative growth. However, yeast also consumes carbon sources such as galactose, which is presumed to cause simultaneous respiration and fermentation [11-13]. Another case is mannose, where levels of the typical carbon catabolite repression reporter Suc2p show that carbon catabolite repression is significantly decreased [14], which would suggest higher respiration than on glucose. How is respiration regulated transcriptionally on these carbon sources?

Here, we address this question quantitatively by linking the functional degree of respiration to transcriptional regulation via enzyme abundances. For this purpose, we used galactose and mannose as examples of potential intermediate respiration, and glucose and pyruvate as extreme cases of minimal and maximal respiration in aerobic batch cultures, respectively. Respiration is quantified as the flux through the TCA cycle by ^13^C flux analysis [15-17]. Protein abundances of respiratory enzymes are quantified with GFP fusion strains [18,19]. From these data we then predicted the most probable transcription factors that control the coordinated differential expression of respiratory enzymes under the various conditions. These predictions were then verified on the basis of functional changes in the corresponding transcription factor deletion strains, leading to a carbon source dependent model of transcriptional regulation of respiration.

## Results and Discussion

### Degree of respiration on different carbon sources

**Figure 1:**
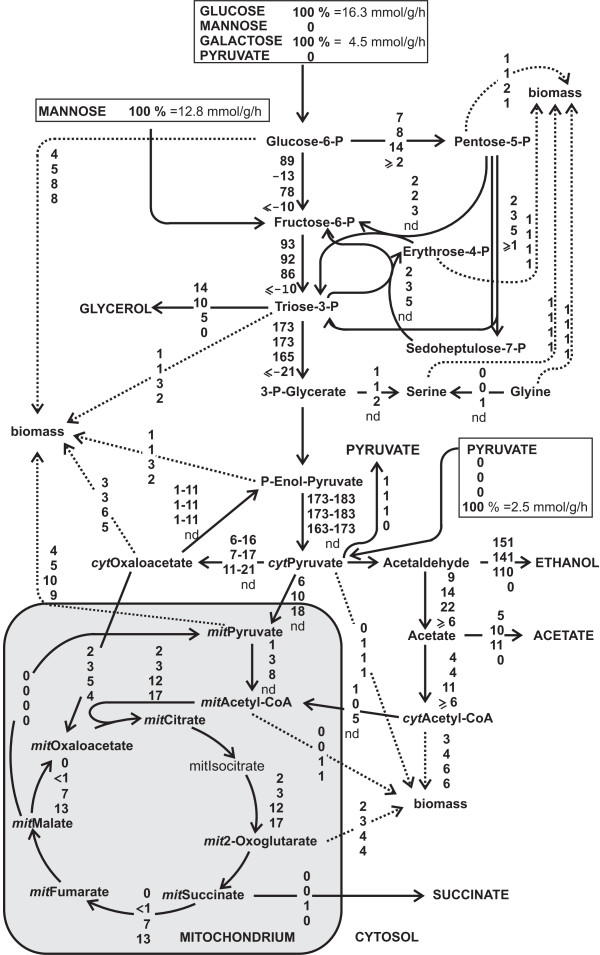
***In vivo *fluxes normalized to substrate uptake rates in exponentially growing batch cultures on glucose (position 1), mannose (position 2), galactose (position 3) and pyruvate (position 4)**. Glucose and galactose fluxes were calculated from separately performed 1-^13^C and U-^13^C labeled substrates, mannose and pyruvate only from U-^13^C labeled substrates. The error on most fluxes is less than 20%. 'nd' stands for not determined.

To elucidate the transcriptional regulation of respiration, we grew wild-type *S. cerevisiae *FY4 in aerobic microscale batch cultures (96-deep-well plates) on minimal medium with either glucose, mannose, galactose or pyruvate as sole carbon source. For a functional quantification of respiration, we determined intracellular carbon fluxes with ^13^C flux analysis in eight independent cultures per carbon substrate [20-22]. For this purpose, yeast was grown on either ^13^C labeled glucose, mannose, galactose or pyruvate. These carbon substrates are degraded via different metabolic pathways that lead to different ^13^C labeling patterns which were subsequently measured in protein-bound amino acids by gas chromatography - mass spectrometry (GC-MS). From the determined mass isotopomer abundances in amino acids, we calculated eight ratios of converging central metabolic fluxes with algebraic equations [21]. These determined flux ratios (additional files 1) were then combined with physiological data (Table 1, additional file 1) such as the carbon uptake rate and a stoichiometric model of central yeast metabolism to fit metabolic fluxes in mmol/g/h to the measured data [23] (Figure 1, additional file 1).

**Table 1:** Physiological data of *S. cerevisiae *FY4 during exponential batch growth on four carbon sources

c-source	uptake rate [mmol/g/h]	biomass yield [g(*cdw*)/g(*c - source*)]	growth rate [1/h]	degree of respiration [*AU*]
glucose	-16.3 ± 1.1	0.11 ± 0.01	0.33 ± 0.01	0.00 ± 0.00
mannose	-12.8 ± 1.1	0.14 ± 0.03	0.32 ± 0.01	0.06 ± 0.00
galactose	-4.5 ± 0.3	0.25 ± 0.02	0.20 ± 0.01	6.90 ± 1.30
pyruvate	-2.5 ± 0.4	0.45 ± 0.04	0.10 ± 0.01	13.30 ± 2.66

Standard deviations were fitted from eight independent cultures. Error ranges for the degree of respiration were fitted with the input of eight independent cultures.

Since the overall rate of metabolism differed widely, as can be seen from the substrate uptake rates (Table 1), we normalized the calculated metabolic fluxes by the uptake rate to enable a direct comparison (Figure 1). The thus normalized fluxes on glucose and mannose were very similarly distributed. On galactose, by-product formation was significantly reduced, while the TCA cycle and biomass fluxes were increased. On pyruvate, yeast exhibited exclusively respiratory metabolism without ethanol formation and, of course, a completely reversed glycolytic flux.

In the following we are primarily interested in a quantitative measure of respiration that would be directly comparable between the conditions. Hence we used the normalized flux from mitochondrial malate to mitochondrial oxaloacetate as a measure for the respiratory flux through the TCA cycle, which we therefore define as the degree of respiration. This degree of respiration was marginal on glucose and mannose, significant on galactose and, as expected, highest on pyruvate where energy production completely relies on respiration. The determined biomass yields and fluxes to ethanol correlated with the degree of respiration, thus confirming the conclusion on the degree of respiratory metabolism of the four carbon sources (Table 1, Figure 1). Our finding on the different metabolic states during growth on the tested carbon sources are also in agreement with previous studies such as the work of Polakis and Bartley [24].

### Protein abundances

The general expectation is that altered levels of respiration are accomplished through modulations in enzyme abundances [1]. To elucidate which of the approximately 60 respiratory enzymes are relevant for the different degrees of respiration, we used protein-GFP fusion strains that are typically used in protein localization studies [18]. To use the fluorescence signal of C-terminally tagged proteins to quantify protein abundances raises the issue whether the fused proteins are still catalytically active, e.g. correctly folded, correctly aggregating into multimers/complexes and still subject of allosteric regulation. While the final proof is not possible, we provide two lines of evidence that the GFP-fusion proteins function normal in the majority of the cases. Firstly, we quantified the specific growth rate as an overall measure of physiological function. On average, 80% of the investigated strains grew at a rate that was indistinguishable of the reference strain under the four tested conditions. Secondly, as a more sensitive and specific measure of functionality, we quantified intracellular concentrations of 43 metabolites in four GFP-fusion strains (*PDA1-GFP::HIS3, IDH1-GFP::HIS3 *(isocitrate dehydrogenase is subject of allosteric regulation), *COX8-GFP::HIS3, QCR6-GFP::HIS3*) and the reference strain during growth on galactose. We found on average a differences of 1.2 fold changes (fold change below 1.3 is not significant) between the metabolite concentrations of fusion and reference strain; only very few metabolites were altered by up to 2 fold changes (data not shown). Collectively, this data indicate that the majority of the GFP-fused proteins are still functional as it was also found by Newman *et. al *[19].

We investigated in total 47 protein-GFP fusion strains, each strain containing one protein-GFP fusion construct. These include 21 enzymes from the TCA cycle, 19 from the respiratory chain and 7 enzymes from the ethanol production pathway (additional file 2). The coverage for TCA cycle, respiratory chain and ethanol production enzymes was more than 84%, 70% (excluding the ATPase) and 78%, respectively. The 47 GFP-tagged strains were grown on the four carbon sources and we determined the steady state enzyme abundances on each of the four carbon sources from online fluorescence and biomass measurements in 96-well plates.

**Figure 2:**
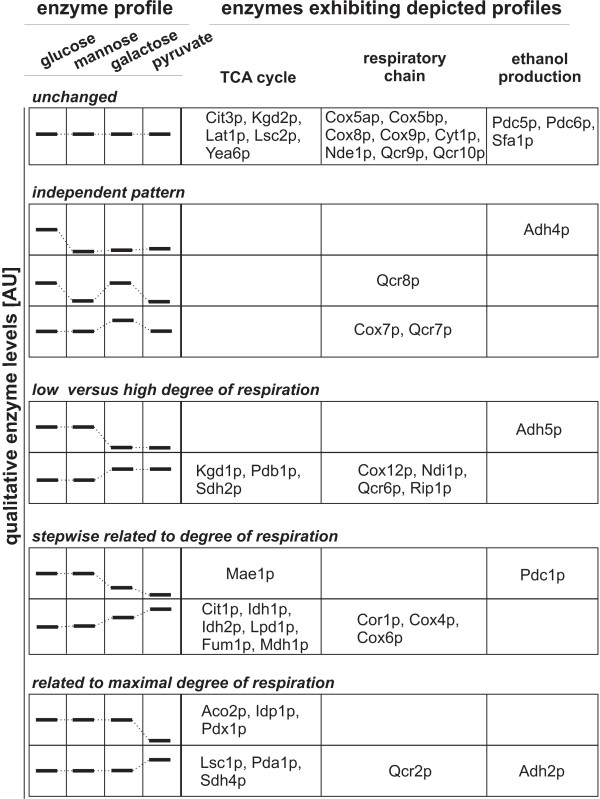
**Carbon source dependent enzyme expression patterns**. Enzyme abundances were determined by on-line fluorescence of GFP-fusion strains during exponential growth on alternative carbon substrates. Enzyme abundances on mannose, galactose and pyruvate were plotted relative to those during growth on glucose. As a function of altered enzyme abundances, the 47 investigated enzymes (21 TCA cycle, 19 respiratory chain and 7 fermentative enzyme) were divided into five major enzyme expression pattern classes.

More than 60% of all investigated enzymes changed their level of expression during growth on at least one carbon source compared to glucose (Table 2). While the expression levels on mannose were almost identical to those on glucose, 20 of 40 investigated enzymes that catalyze TCA cycle and the respiratory chain reactions were up-regulated on galactose and/or pyruvate and only five enzymes were down-regulated. The GFP-fusion strains of *RIP1 *and *COX12 *did not grow on galactose and pyruvate, very likely because the fusion enzymes were not functional anymore. Thus, Rip1p and Cox12p were considered as important for respiration. Next we looked for patterns of enzyme expression that are related to respiration and whether these would be over-represented in one of the enzyme categories that we investigated (Figure 2). There was clearly an over representation of TCA cycle enzymes (70% of all investigated TCA cycle enzymes) in enzyme expression patterns that were positively related with respiration (Figure 2). Of all respiratory chain enzymes in contrast only 42% were found to be positively related with respiration (Figure 2). For each reaction in the TCA cycle there was at least one catalyzing enzyme up-regulated under respiratory metabolism with the exception of succinyl-CoA ligase for galactose. For the aconitase catalyzed reaction we had no information about the main enzyme Aco1p since it was not available as GFP-fusion. The isoenzyme Aco2p was negatively related with respiration, as expected, since it is induced on glucose and repressed on non-fermentable carbon sources [25,26]. In the respiratory chain there was on galactose and pyruvate at least one subunit per complex (succinate dehydrogenase, cytochrome bc1, cytochrome c oxidase) up-regulated, while for the NADH dehydrogenase only the internal (Ndi1p) but not the external one (Nde1p) was increased in expression.

**Table 2:** Fold changes in metabolic enzyme expression

**enzyme**	**man**	**gal**	**pyr**	**enzyme**	**man**	**gal**	**pyr**
	
***TCA cycle***				***respiratory chain without ATPase***			
Yea6p	nsc	nsc	nsc	Ndi1p	nsc	2.4	2.1*
Pda1p	nsc	nsc	2.8	Nde1p	nsc	nsc	nsc
Pdb1p	nsc	3.0	2.7	Cyt1p	nsc	nsc	nsc
Lat1p	nsc	nsc	nsc	Cor1p	nsc	3.8	6.0
Lpd1p	nsc	3.1	5.1*	Qcr2p	nsc	nsc	5.9
Pdx1p	nsc	nsc	0^‡^	Qcr6p	nsc	3.7	2.8*
Cit1p	nsc	6.0	14.1	Qcr7p	nsc	2.5	nsc
Cit3p	nsc	nsc	nsc	Qcr8p	0.2*	nsc	0.2
Aco2p	nsc	nsc	0.3	Qcr9p	nsc	nsc	nsc
Idh1p	nsc	2.1	3.0	Qcr10p	nsc	nsc	nsc
Idh2p	nsc	2.5	3.6	Rip1p	nsc	ng	ng
Idp1p	nsc	nsc	0.6	Cox4p	nsc	3.6*	5.1
Kgd1p	nsc	2.7	2.9	Cox5ap	nsc	nsc	nsc
Kgd2p	nsc	nsc	nsc	Cox5bp	nsc	nsc	nsc
Lsc1p	nsc	nsc	2.5	Cox6p	nsc	3.9	5.8*
Lsc2p	nsc	nsc	nsc	Cox7p	nsc	5.0	nsc
Sdh2p	0^‡^	4.8	4.6**	Cox8p	nsc	nsc	nsc
Sdh4p	nsc	nsc	2.8*	Cox9p	nsc	nsc	nsc
Fum1p	nsc	3.1	6.8	Cox12p	nsc	ng	ng
Mdh1p	nsc	3.9	7.4*	***ethanol production***			
Mae1p	nsc	0.4**	0^‡^	Pdc1p	0.9	0.6	0.4
				Pdc5p	nsc	nsc	nsc
nsc = not significantly changed				Pdc6p	nsc	nsc	nsc
ng = no growth				Adh2p	nsc	nsc	16.9
0^‡ ^= under detection limit				Adh4p	0*‡	0.1*	0.2*
* = p-value 0.06-0.10				Adh5p	nsc	0.1	0.1
** = p-value 0.11-0.12				Sfa1p	nsc	nsc	nsc

Fold changes in enzymes expressed on mannose (man), galactose (gal) or pyruvate (pyr) compared to growth on glucose (glc) (alternative carbon source/glucose). Expression levels are determined during exponential growth in batch with strains containing a single enzyme-GFP construct. Significant changes were assigned when the p-value was ≤ 0.12 (calculated from three independent experiments) compared to the enzyme expression during growth on glucose.

**Figure 3:**
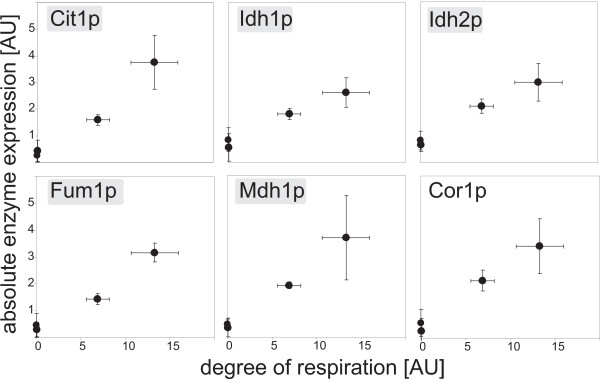
**Respiratory enzymes whose abundance alteration exhibited a significant correlation to the degree of respiration**. Significance for a correlation between enzyme levels and the corresponding degree of respiration on the four carbon sources was assigned by correlation factors of 0.90 or greater. Enzymes highlighted in grey are TCA cycle enzymes, Cor1p is a component of the respiratory chain.

To elucidate a potential quantitative correlation between enzyme levels and degree of respiration, we plotted the measured abundance for each enzyme against the corresponding degree of respiration and calculated the correlation factor (data not shown). Six respiratory enzyme level - degree of respiration curves exhibited a correlation factor of 0.90 or higher (Figure 3), additionally correlated Pdc1p, an enzyme from the ethanol production pathway, negatively with the degree of respiration. These findings are consistent with increased *in vitro *enzyme activities of Idh1/2p, Fum1p and Mdh1p on galactose and pyruvate compared to glucose [24]. In our data set again, enzymes of the TCA cycle were over represented in the set of respiratory enzymes that correlate with the degree of respiration. We thus conclude that mainly the TCA cycle is important for establishing the different degrees of respiration under the tested conditions. While the respiratory chain is certainly also required, all of its expression levels except of those of Cor1p do not correlate with the degree of respiration and we have no evidence for a carbon source dependent coordinated up-regulation of all subunits of the respiratory chain.

### Transcriptional regulation of respiration

**Figure 4:**
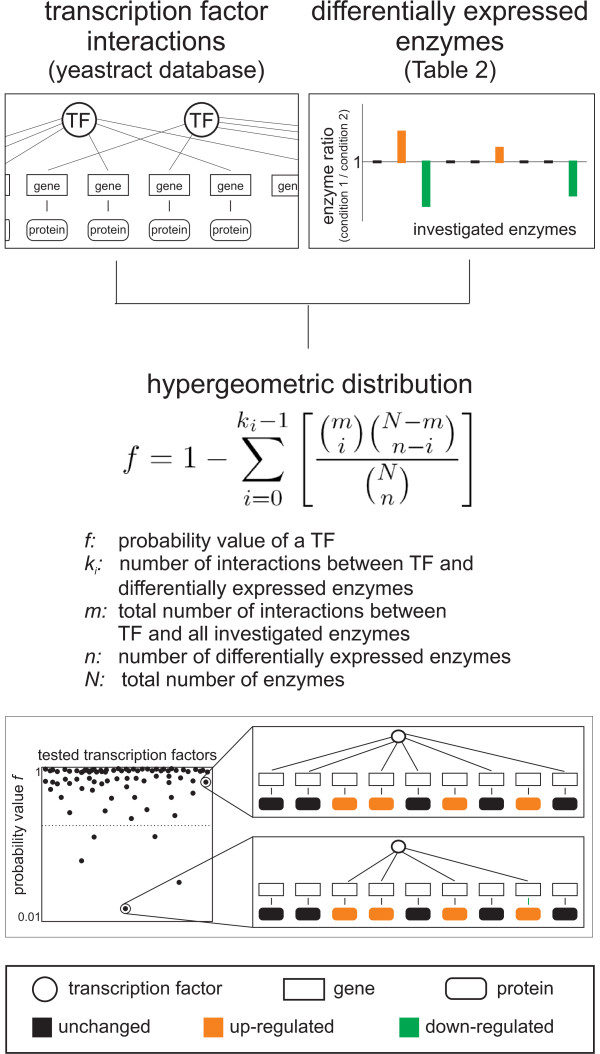
**Statistical inference of involved transcription factors**. Schematic overview of prediction of transcription factors that potentially regulate differential expressed enzymes. The calculated *f *value gives the probability for the target pattern of a transcription factor to include by chance the identified differential expressed enzymes. Thus, transcription factors with low probability value *f *are likely to regulate the differential expressed enzymes.

Since transcriptional regulation is the most probable mechanism to achieve these distinct modulations in respiratory enzyme abundances, we were interested to identify the involved transcription factors. For this purpose we applied a statistical framework that evaluates the probability of each transcription factor to be responsible for the observed differential expression pattern. Specifically, we used the pairwise fold changes between enzyme levels on galactose and pyruvate compared to those on glucose; i.e. 18 and 21 enzymes were up-regulated on galactose and pyruvate compared to glucose, respectively (Table 2). From the yeastract database [27], we then selected all transcription factors that had at least one target (literature curated) among the 47 investigated enzymes. Our statistical analysis is based on the hypothesis that a transcription factor with the same target genes as the observed pattern of differential expressed enzymes has a high probability to be responsible for causing the change (Figure 4). Transcription factors that include the differentially expressed enzymes in their target pattern, but have many additional targets that were not differentially expressed in our data set, in contrast, have a low probability to be responsible. Specifically, we calculated with a hypergeometric distribution a probability value *f*, that the target pattern of a transcription factor by chance includes the pattern of measured differential expressed enzymes [28,29] (Figure 4). At low probability values *f*, the considered transcription factor is likely to be involved in the regulation of the observed differential expressed enzymes. Since the biological meaning of the absolute value of *f *is limited, we considered always the transcription factors with the five lowest *f *values as relevant.

**Figure 5:**
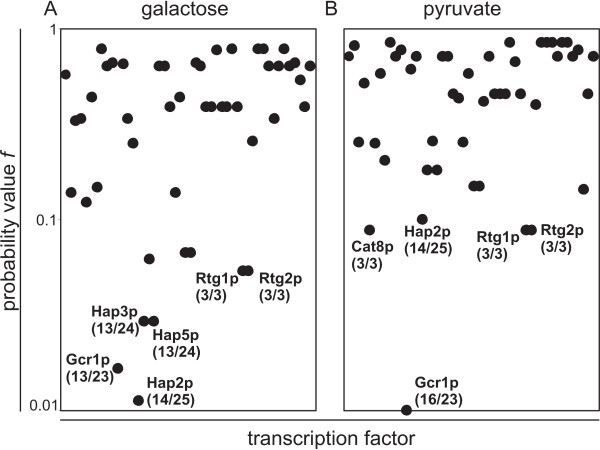
**Probability value *f *calculated from enzyme expression patterns on galactose (A) and pyruvate (B)**. The calculated *f *value gives the probability for the target pattern of a transcription factor to include by chance the identified differential expressed enzymes. First number in parenthesis gives *k*_*i*_, second number in parenthesis gives *m *for each potentially regulating transcription factor.

On galactose compared to glucose, we identified the transcription factors Gcr1p, Hap2p, Hap3p, Hap5p, Rtg1p and Rtg2p as most likely to be responsible for the observed up-regulated enzymes (Figure 5A). On pyruvate compared to glucose, we identified the transcription factors Cat8p, Gcr1p, Hap2p, Rtg1p and Rtg2p (Figure 5B). For the Hap-complex, this result was expected since it is a global activator complex of respiration [1,4,6-8]. The Rtg-proteins are only known as activators of some TCA cycle genes in cells with compromised mitochondrial function [4-6], beside their function in retrograde signaling. Cat8p activates the gluconeogenic and glyoxylate cycle genes *PCK1, FBP1, ICL1 *and *MLS1 *[3,4,9,10]. Furthermore, it was shown that *HAP4 *activation depends on an intact *CAT8 *gene [4]. The main known function of Gcr1p is the activation of glycolysis genes [30]. From these results we hypothesize that the above transcription factors influence respiration through activation (Cat8p, Hap2/3/5p and Rtg1/2p) or repression (Gcr1p) of their respiratory target genes.

**Figure 6:**
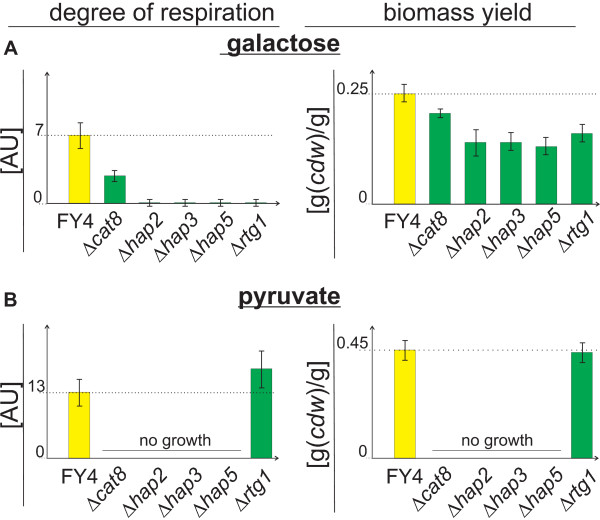
**Verification of predicted respiration relevance of transcription factors based on deletion strains**. Degree of respiration and biomass yield in transcription factor deletion strains grown exponentially on galactose (A) or pyruvate (B).

To verify this hypothesis, we grew deletion mutants of the predicted *HAP*, *RTG1 *and *CAT8 *transcription factors on galactose and pyruvate. We determined the biomass yield and the normalized respiratory TCA cycle flux by ^13^C flux analysis as functional measures of the degree of respiration (Figure 6, Table 3). When a predicted transcription factor is indeed responsible for the level of respiration observed in the wild-type strain on galactose or on pyruvate, the degree of respiration and the biomass yields should be decreased in the corresponding deletion mutant.

**Table 3:** Biomass yield and degree of respiration for deletion strains of transcription factors that were predicted to regulate respiration

c-source	deletion strain	biomass yield [g(*cdw*)/g(*c - source*)]	degree of respiration [*AU*]
galactose	FY4	0.25 ± 0.02	6.9 ± 1.3
	Δ*cat8*	0.21 ± 0.01	2.8 ± 0.6
	Δ*hap2*	0.14 ± 0.03	0.0 ± 0.2
	Δ*hap3*	0.14 ± 0.02	0.0 ± 0.2
	Δ*hap5*	0.13 ± 0.02	0.0 ± 0.2
	Δ*rtg1*	0.16 ± 0.02	0.0 ± 0.2

pyruvate	FY4	0.45 ± 0.04	13.3 ± 2.7
	Δ*cat8*	no growth	
	Δ*hap2*	no growth	
	Δ*hap3*	no growth	
	Δ*hap5*	no growth	
	Δ*rtg1*	0.44 ± 0.04	18.1 ± 3.6

Biomass yield standard deviations were calculated from at least two independent cultures. Error ranges for the degree of respiration were fitted with the input of at least two independent cultures.

The *CAT8 *deletion strain did not grow on pyruvate as expected, because Cat8p is essential for the activation of gluconeogenic genes [9,31]. On galactose, where gluconeogenesis is not required the *CAT8 *deletion strain exhibited a significantly decreased biomass yield and degree of respiration compared to the wild-type, confirming its importance for respiration. Yet, Cat8p is not essential for respiration, since respiration is not completely absent in the *CAT8 *deletion strain on galactose (Table 3, Figure 6). The importance of Cat8p for respiration is thus independent from its function in gluconeogenesis. This might be explained by a requirement of Cat8p for full Hap4p activation or the necessity of Cat8p activation of its TCA cycle targets *IDH1 *and *FUM1 *[27]. For all *HAP *deletion strains, the degree of respiration and the biomass yield were indeed significantly lower than in the wild-type strain during growth on the partly respired substrate galactose (Table 3, Figure 6). On the non fermentable substrate pyruvate, the *HAP *deletion strains were not able to grow. The respiratory phenotype of the *RTG1 *deletion strain on galactose was basically identical to those of the *HAP *deletion strains (Table 3, Figure 6). On pyruvate, in contrast, the *RTG1 *deletion strain exhibited basically a wild-type like biomass yield and degree of respiration. While the finding for *RTG1 *deletion strain grown on pyruvate fits literature data and models [32,33], our finding for *RTG1 *deletion strain grown on galactose was unexpected because the literature model [33] ascribes Rtg1p activity to impaired mitochondrial function as in [*rho*^0^] cells, which is certainly not the case for growth on galactose.

## Conclusion

We quantified the proportion of respiratory energy generation on four differentially repressive carbon sources and identified, by combined experimental and computational analysis, key transcription factors that are important for respiration in *S. cerevisiae*. We confirmed in this study the essential role of Hap2/3/5p in respiration, identified essentiality of Rtg1p for respiration on galactose and identified an important, but not essential role for Cat8p in attaining a wild-type like degree of respiration. This Cat8p-based regulation of respiration is independent of the known regulation of gluconeogenesis because the *CAT8 *mutant grows well on the glycolytic substrate galactose, but with significantly reduced respiration.

**Figure 7:**
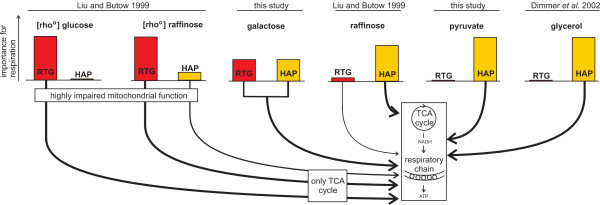
**Carbon source and mitochondrial function dependent model of transcriptional regulation of respiration**. Expansion of the initial model of Liu and Butow [33] that was solely mitochondrial function dependent. Line thickness indicates importance for respiration.

While importance of the Hap-complex for respiration was known [1,4,6-8], the impact of the Rtg-proteins on respiration cannot be solely explained by the previously proposed model that ascribes their activity to cells with highly impaired mitochondrial function [33] or by their stress induced role in retrograde signaling [34]. Thus, we propose an expanded model which adds the aspect of carbon source, and thus the degree of respiration dependent activity pattern, for the Rtg-proteins and the Hap-complex (Figure 7). The newly added aspect of the carbon source is even seen for the activity pattern of the Hap-complex in [*rho*^0^] cells with highly impaired mitochondrial function during growth on either glucose or raffinose (Figure 7). This study provides further knowledge on the regulation of respiration on four differentially repressive carbon sources by combining the transcription factor target network with experimentally measured protein expression levels using a statistical framework.

## Methods

### Strains, medium and cultivation conditions

The strains used in this study are listed in Table 4. All liquid cultivations were carried out using minimal medium as described in Blank and Sauer (2004) [21]. The pre-cultures were always cultured in glucose minimal medium. Other carbon sources or labeled substrates were only added to the experiment culture at 10 g/l each.

**Table 4:** Strains used in the study

experiment	strains and relevant genotype	source
degree of respiration	rel. genotype: FY4 MAT**a **FY4	[41]

enzyme expression	rel. genotype: BY4741 MAT**a ***ura3*Δ*0, leu2*Δ*0, his3*Δ*1, met15*Δ*0*	Open-biosystems
	*PDC1-GFP::HIS3, PDC5-GFP::HIS3, PDC6-GFP::HIS3*,	
	*ADH4-GFP::HIS3, ADH5-GFP::HIS3, SFA1-GFP::HIS3*,	
	*YEA6-GFP::HIS3, PDA1-GFP::HIS3, PDB1-GFP::HIS3*,	
	*LAT1-GFP::HIS3, LPD1-GFP::HIS3, PDX1-GFP::HIS3*,	
	*CIT1-GFP::HIS3, CIT3-GFP::HIS3, ACO2-GFP::HIS3*,	
	*IDH1-GFP::HIS3, IDH2-GFP::HIS3, IDP1-GFP::HIS3*,	
	*KGD1-GFP::HIS3, KGD2-GFP::HIS3, LSC1-GFP::HIS3*,	
	*LSC2-GFP::HIS3, SDH2-GFP::HIS3, SDH4-GFP::HIS3*,	
	*FUM1-GFP::HIS3, MDH1-GFP::HIS3, MAE1-GFP::HIS3*,	
	*NDI1-GFP::HIS3, NDE1-GFP::HIS3, CYT1-GFP::HIS3*,	
	*COR1-GFP::HIS3, QCR1-GFP::HIS3, QCR2-GFP::HIS3*,	
	*QCR6-GFP::HIS3, QCR7-GFP::HIS3, QCR8-GFP::HIS3*	
	*QCR9-GFP::HIS3, RIP1-GFP::HIS3, COX4-GFP::HIS3*,	
	*COX5A-GFP::HIS3, COX5B-GFP::HIS3, COX6-GFP::HIS3*,	
	*COX7-GFP::HIS3, COX8-GFP::HIS3, COX9-GFP::HIS3*,	
	*COX12-GFP::HIS3, HO-TAP::HIS3*	

transcriptional regulation	rel. genotype: FY4 MAT**a**	[41], C. Boone
	Δ*cat8*, Δ*hap2*, Δ*hap3*, Δ*hap5*, Δ*rtg1*, Δ*FY4*	

For flux analysis experiments, FY4 was freshly plated from a glycerol stock on a YPD (1% yeast extract, 2% peptone and 2% glucose) plate. The liquid pre-culture was inoculated from the YPD plates. For the experiment cultures, minimal medium containing 10 g/l of either glucose, mannose, galactose or pyruvate as sole carbon source was used. FY4 was cultured in 96-deep-well plates (Kuehner AG, Birsfeld, Switzerland) [35] as batch cultures at 30°C and 300 rpm in a shaker with 50 mm shaking amplitude. The culture volume was 1.2 ml. To improve mixing, a single 4 mm diameter glass bead (Sigma-Aldrich, Buchs, Switzerland) was added per deep-well. The minimal medium for experiment cultures contained a mixture of 20% [U-^13^C] labeled glucose (^13^C enrichment ≥ 99%, Cambridge Isotope Laboratories, Andover, USA) and 80% naturally labeled glucose. The same experiment was performed separately for mannose (^13^C enrichment ≥ 99%, Omicron Biochemicals, South Bend, USA), galactose (^13^C enrichment ≥ 98%, Omicron Biochemicals, South Bend, USA) and pyruvate (^13^C enrichment ≥ 99%, Cambridge Isotope Laboratories, Andover, USA). For glucose and galactose, additional flux experiments were performed with 100% [C1-^13^C] labeled glucose (^13^C enrichment ≥ 99%, Cambridge Isotope Laboratories, Andover, USA) or galactose (^13^C enrichment ≥ 99%, Omicron Biochemicals, South Bend, USA) to better resolve the pentose phosphate pathway.

For the protein expression experiments, GFP-tagged strains and the TAP-tagged reference strain were plated from glycerol stocks on minimal medium plates supplemented with leucine (0.24 g/l), methionine (0.115 g/l) and uracil (0.05 g/l). Liquid pre-cultures of minimal medium containing leucine (0.24 g/l), methionine (0.115 g/l) and uracil (0.05 g/l) were inoculated from the minimal medium plates with 10 g/l glucose. The pre-cultures were cultivated in 96-deep-well plates with the same conditions as explained above. For the experimental culture, additionally histidine (0.025 g/l) was added to the minimal media already containing leucine, methionine and uracil, the carbon source was either 10 g/l glucose, mannose, galactose or pyruvate. The experiment cultures were cultivated in 96-microtiterplates in an incubator with online monitoring of biomass and fluorescence signals (mp2-labs, Aachen, Germany) at 30°C and 800 rpm and a shaking diameter of 3 mm.

For the transcriptional regulation experiment, the transcription factor deletion strains were freshly plated from glycerol stocks on YPD plates containing 300 μg/ml geneticin (G418) (Gibco, Paisley, UK). The liquid pre-cultures were inoculated from the YPD plates. For the liquid cultures yeast minimal medium was used. The pre-cultures were cultivated in 96-deep-well plates with 10 g/l glucose as described above. Medium and cultivation conditions were identical to the flux experiment described above. Only [U-^13^C] experiments were performed.

### Determination of growth rate, uptake and secretion rates

Growth rates were determined in eight independent experiments on the naturally labeled carbon sources. To determine the growth rate, the optical density at a wavelength of 600 nm was measured in a spectra-photometer (Molecular Devices, Sunnyvale, USA) for 8 to 12 times over the whole growth curve of FY4. Specific growth rates were determined by linear regression of the logarithmic OD_600 _values over time from at least 6 data points at maximum rate.

The supernatant samples from mid-exponential growing cells were analyzed with an HPX-87H Aminex, ion-exclusion column (Biorad, Munchen, Germany) as described previously [36,37] on an HPLC HP1100 system (Agilent Technologies, Santa Clara, USA). The column temperature was 60°C and as eluant 5 mM H_2_SO_4 _was used with a flow rate of 0.6 ml/min. Pyruvate, succinate and acetate were determined at a wavelength of 210 nm with the UV detector. Glucose, mannose, galactose, glycerol and ethanol were measured with a refractive index detector. The uptake and secretion rates were determined from two points (beginning of exponential growth and mid exponential growth), in eight replicates. The substrate or by-product concentrations during exponential growth were plotted against the corresponding cell dry weights. The cell dry weights were calculated from the OD_600 _values multiplied with a conversion factor that was previously determined. A linear fit was applied to calculate the slope. The inverse of this slope is the biomass yield in g/mmol. The non inversed slope was further multiplied with the growth rate to get uptake and secretion rates.

### Flux analysis

The labeled cultures were inoculated with an OD_600 _of 0.015 or less. 1 ml of culture was harvested during mid-exponential growth (OD_600 _0.5 - 1.2). The cells were washed three times with ddH_2_O and stored at -20°C for GC-MS analysis. The supernatant was stored for determining uptake and secretion rates of glucose, mannose, galactose, pyruvate, ethanol, acetate, glycerol and succinate at -20°C. The experiment was repeated at least two times.

Samples for GC-MS analysis were prepared as described previously [20]. The frozen cell pellet was hydrolyzed with 6 mol/l HCl for 12 h at 105°C. The samples were dried at 95°C under a constant air stream. They were derivatized using 20 μl of the solvent DMF (Sigma-Aldrich, Buchs, Switzerland) and 20 μl of the derivatization agent *N-(tert*-butyldimethylsilyl)-*N*-methyl-trifluoroacetamide with 1% *tert*-butyldimethylchlorosilane (Sigma-Aldrich, Buchs, Switzerland) for 1 h at 85°C. The mass isotopomer distributions of the protein-bound amino acids were measured with a 6890N GC system (Agilent Technologies, Santa Clara, USA) combined with a 5973 Inert XL MS system (Agilent Technologies, Santa Clara, USA).

Flux ratios were determined from the mass isotopomer distribution of the protein-bound amino acids with the software FiatFlux [38] using the analytical equations developed by Blank and Sauer (2004) [21]. For the determination of the TCA cycle flux ratio, equation 3 of Blank and Sauer (2004) was applied [21]. For growth on pyruvate, only the split between anaplerosis and the TCA cycle could be resolved. The mass isotopomer distribution was corrected for the amount of unlabeled biomass and naturally occurring stables isotopes [20]. For the calculation of net fluxes, all flux ratios were taken from the [U-^13^C] experiment except for the carbon sources glucose and galactose where the ratio for the split between glycolysis and the pentose phosphate pathway was obtained from a [C1-^13^C] experiment. The gluconeogenic ratio varied dependent on the tuning of the MS instrument therefore a flux ratio range was used for the later calculation.

Net fluxes [39,23] were calculated with the software Fiat Flux. The stoichiometric equation system was solved with constraints of ratios, uptake, secretion and biomass formation rates. For growth on pyruvate, only the flux through the TCA cycle was calculated from the split ratio between anaplerosis and the respiratory TCA cycle. The anaplerotic fraction of the flux into the TCA cycle equals the flux from 2-oxoglutarate to the biomass. Since Mae1p is not expressed during growth on pyruvate (enzyme abundance measurement) we can calculate the actual flux through the TCA cycle from the flux through the anaplerotic reaction and the flux ratio. For all further calculations we assume a standard deviation of 20% for the respiratory flux through the TCA cycle on pyruvate. In the same way then described for pyruvate, the respiration is quantified in the transcription factor deletion strains.

### Protein abundance measurement

The enzyme expression level was calculated from the slope between the biomass signal (light scattering) [40] (excitation at 620 nm) and the GFP signal (excitation at 486 nm, emission at 510 nm) gained from the protein GFP-fusion strains [18] (Table 4). *HO-TAP::HIS3 *was used as reference to correct for auto fluorescence. Thus, the slope between the GFP and the biomass signal, calculated for the reference was subtracted from the slope calculated for the GFP-fusion strains. The slope, corrected for the autofluorescence, quantifies the enzyme abundances. Significant changes are assigned for p-values of 0.12 or lower which ensures that the error ranges between enzyme expression during growth on glucose compared to each other carbon source do not overlap and thus truly differential expressed enzymes are further investigated.

### Prediction of involved transcription factors

Transcription factors are more often associated with a subset of differentially expressed proteins than expected by chance were determined by a statistical analysis adopted from Boyle *et al*. [28] as outlined in Kümmel *et al*. [29]: The likelihood is calculated with the hypergeometric distribution that a transcription factor is associated with the differential expressed enzymes between two conditions compared to all enzymes (Figure 4).

*f *: probability value of a transcription factors

*k*_*i*_: number of interactions between a transcription factor and the differential expressed enzymes

*m *: total number of interactions between a transcription factor and all investigated enzymes

*n *: number of differential expressed enzymes

*N *: total number of enzymes

The such calculated significants value *f *gives the probability that the transcription factor has at least the observed number of interactions with the subset of differential expressed enzymes by chance. When the probability value *f *is low we conclude that the considered transcription factor is likely to be involved in the regulation of the observed differential expressed enzymes. The transcription factor interactions were from the yeastract database (literature curated once) [27]. The differential expressed enzymes were in this study determined with GFP-fusion strains [18], whereas only p-values of 0.12 or lower were assigned as differentially expressed, which ensures that the error ranges between enzyme expression during growth on glucose compared to each other carbon source do not overlap and thus truly differential expressed enzymes are further investigated. We assigned transcription factors being most likely to regulate the up-regulated enzymes based on the *f *value (Figure 5). Thus we used, as compromise between the two data sets, the transcription factors with the lowest five *f *values for the further analysis. The program code is written in MatLab.

## Abbreviations

AU: arbitrary units; *cdw*: cell dry weight; CoA: coenzyme A; *cyt*: cytosolic; dd: double distilled; DMF: dimethyl-formamide; gal: galactose; GC: gas chromatograph; glc: glucose; man: mannose; *mit*: mitochondrial; MS: mass spectrometry; OD: optical density; pyr: pyruvate; TCA: tri-carboxylic-acid; UV: ultra violet.

## Authors' contributions

SMF designed the study, performed all experiments and drafted the manuscript. US conceived and supervised the study and revised the manuscript. All authors read and approved the final manuscript.

## Supplementary Material

Additional file 1 **Ratios and net fluxes**. Flux rations and absolute metabolic (net) fluxes for yeast grown on either, glucose, mannose, galactose or pyruvate. Click here for file

Additional file 2 **Function of investigated enzymes**. Description of the functions of all investigated enzymes as listed on http://www.yeastgenome.org. Click here for file
